# Insights into Ligand Binding to PreQ_1_ Riboswitch Aptamer from Molecular Dynamics Simulations

**DOI:** 10.1371/journal.pone.0092247

**Published:** 2014-03-24

**Authors:** Zhou Gong, Yunjie Zhao, Changjun Chen, Yong Duan, Yi Xiao

**Affiliations:** 1 Biomolecular Physics and Modeling Group, Department of Physics, Huazhong University of Science and Technology, Wuhan, Hubei, China; 2 Genome Center and Department of Biomedical Engineering, University of California Davis, Davis, California, United States of America; Oak Ridge National Laboratory, United States of America

## Abstract

Riboswitches play roles in transcriptional or translational regulation through specific ligand binding of their aptamer domains. Although a number of ligand-bound aptamer complex structures have been solved, it is important to know ligand-free conformations of the aptamers in order to understand the mechanism of specific binding by ligands. In this paper, preQ_1_ riboswitch aptamer domain from *Bacillus subtilis* is studied by overall 1.5 μs all-atom molecular dynamics simulations We found that the ligand-free aptamer has a stable state with a folded P1-L3 and open binding pocket. The latter forms a cytosine-rich pool in which the nucleotide C19 oscillates between close and open positions, making it a potential conformation for preQ_1_ entrance. The dynamic picture further suggests that the specific recognition of preQ_1_ by the aptamer domain is not only facilitated by the key nucleotide C19 but also aided and enhanced by other cytosines around the binding pocket. These results should help to understand the details of preQ_1_ binding.

## Introduction

Riboswitches are genetic regulatory elements found in the 5′-untranslated regions of messenger RNA [1]–[5] and are widely distributed in bacteria [6]–[8]. They regulate gene expression or translation through the binding of specific ligand (metabolite), such as adenine [9], guanine [7], 7-aminomethyl-7-deazaguanine (preQ_1_) [10], etc. A riboswitch usually consists of two parts: an aptamer domain and an expression platform. The former specifically binds a metabolite when the concentration of the metabolite exceeds a threshold and the latter regulates the transcription or translation processes through their conformational changes induced by ligand binding. The mechanisms of the specific binding and induced regulation are of great interest [11]–[15]. Recently, the tertiary structures of many riboswitch aptamers have been solved [16]–[18], but almost all of them are ligand-bound complexes and only few of them are in ligand-free states [19]–[23]. This limits our understanding of specific binding mechanisms of the ligands [24].

**Figure 1:**
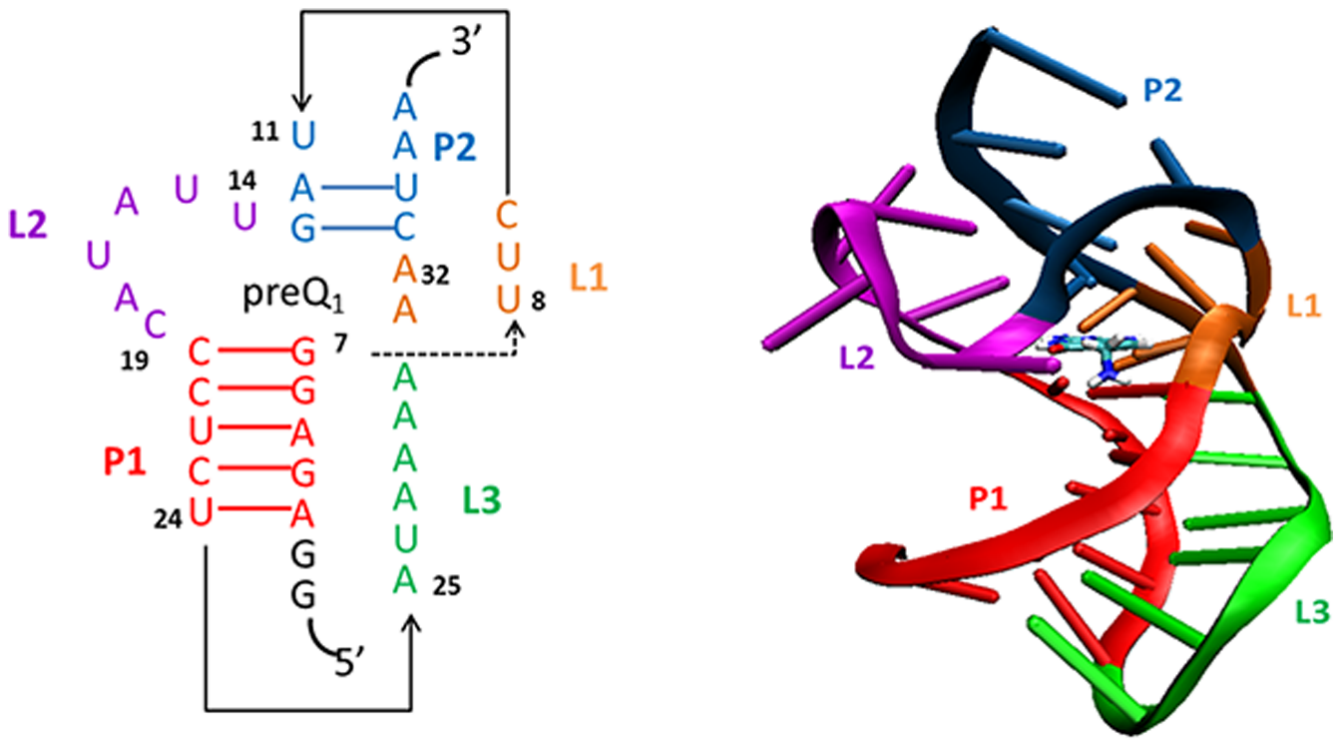
Secondary and tertiary structures of the preQ_1_ riboswitch (PDB ID: 2L1V). The bound preQ_1_ is depicted by a licorice representation. The different parts (P1, P2, L1, L2, and L3) are color-coded.

The modified nucleotide queuosine (Q) is almost universally found in the wobble positions of GUN anticodons in specific tRNAs such as tRNA^His^, tRNA^Asn^, and tRNA^Tyr^ [25]. PreQ_1_ (7-amminomethyl-7-deazaguanine) is a biosynthetic precursor of queuosine synthesized de novo from GTP via a complex series of reactions [26]. In many bacteria, the 5′-UTR of genes associated with synthesis of preQ_1_ have a conserved sequence that has been identified as a class I preQ_1_ riboswitch. The class I preQ_1_ riboswitch contains the smallest known natural aptamer domain, consisting minimally of 36 nucleotides. Kang et al. [27] recently reported the NMR solution structure of the class I preQ_1_ riboswitch aptamer domain from *Bacillus subtilis* (*Bsu*) bound to preQ_1_. They found that the RNA aptamer folds into a generalized H-type pseudoknot with two stems and three loops upon ligand binding, which has not been observed in other RNAs (Fig. 1). Klein and coworkers [28] also identified the cocrystal structure of class I preQ_1_ riboswitch bound to preQ_1_ using X-ray diffraction, which reveals a previously unrecognized pseudoknot fold. However, the knowledge of structural organization of ligand-free aptamer that is crucial to understand the special binding of the ligand is still limited at present. Liberman et al. [23]successfully obtained the crystal structure of a ligand-free aptamer of the preQ_1_ riboswitch from *Thermoanaerobacter tengcongensis* (*Tte*) and found it is very close to that of the ligand-bound state with the adenosine (A14) in the ligand-binding pocket palying the role of preQ_1_. Santner et al.[29] found by using NMR spectroscopy that the free aptamer of the preQ1 class I riboswitch from *Fusobacterium nucleatum* (*Fnu*) preorganizes into a pseudoknot fold, which is close to the ligand-bound state and may be the conformation that.is initially recognized by the ligand. In a previous paper [30] we studied the global unfolding behaviors of two types of preQ_1_ riboswitch aptamer domains by using high-temperature unfolding molecular dynamics (MD) simulations and found stable intermediate states of ligand-free aptamers of both *Bsu* and *Tte* preQ_1_ riboswitches. Very recently, Suddala et al.[31] showed, combining smFRET and NMR with coarse-grained simulations, that the ligand-free aptamers of both *Bsu* and *Tte* preQ_1_ riboswitches have similar ensemble of pre-folded conformations wherein their A-rich 3′ tail adopts transient interactions with the P1-L1 stem-loop, which are different from their ligand-bound structures, They also showed that these pre-folded conformations may be recognized by the ligand, at least for the *Tte* preQ_1_ riboswitch aptamer. These studies suggested possible mechanism of specific ligand binding.

In this paper we further investigate the mechanisms of the ligand binding of the *Bsu* preQ_1_ riboswitch aptamer domain using all-atom molecular dynamics (MD) simulations in explicit solvation. Our results show that the ligand-free preQ_1_ riboswitch aptamer has a stable conformation with P1-L3 structure but an opened binding pocket, which may be a possible candidate for specific binding by the preQ_1_.

## Methods

### MD simulations

Equal number (two 75 ns and one 600 ns) of simulations on the preQ1-bound and free aptamer structures was performed with identical simulation protocol and force fields for comparison. Both preQ1-bound and unbound simulations started from the same experimental NMR bound structure (the first of PDB code 2L1V [27]) whereas the preQ_1_ ligand was removed in the unbound simulations. Each simulation has different initial atomic velocities. All energy minimization and MD simulations were carried out using the *pmemd* program in AMBER 11 software package [32]. Amber ff98 force field was used in these simulations and the solvent was represented explicitly by TIP3P model [24], [33], [34].

Parameters of preQ_1_ were obtained from the Generalized Amber Force Field (GAFF) [35] and the charges were calculated using ANTECHAMBER[36] and AM1-bcc model [37]. The starting structures for simulation were prepared using the TLEAP module in AMBER 11 [32]. The RNA molecule was solvated in an truncated octahedral box containing TIP3P [33] water molecules, with an 8 Å padding in all directions. Charge neutrality in the simulations was achieved by adding 0.15 M (18) Mg^2+^ ions since magnesium ions are essential for the ligand binding as well as the stability of riboswitch [38]. The Amber-adapted van der Waals radius and well depth of magnesium ions are 0.7926 Å and 0.8947 kcal/mol, respectively [39].

Before the MD simulations, the entire systems were first minimized for 1000 steps by steepest descent method followed by 3000 steps of conjugate gradient optimization. During the minimization progress, harmonic forces (500 kcal/mol/Å^2^) were used to restrain the RNA atoms to the experimental positions and water and ions were minimized without restraints. After this stage, the entire system was minimized for 6000 steps without restraint.

In MD simulation stage, the systems were gradually heated up from 0 K to the target temperature 300 K in 200 ps with constant volume during which the RNA molecules were restrained by a harmonic potential (50 kcal/mol/Å^2^) to the initial positions. Then, the restraints on RNA were removed and the simulations were continued under constant pressure that was controlled to 1 bar with a coupling time of 2 ps. The time step was 2 fs and SHAKE [40] algorithm was used to constrain the bonds connecting hydrogen atoms. The Langevin thermostat was used to control the temperature using a collision frequency of 1.0/ps [41]. The long-range electrostatics was treated with the Particle Mesh Ewald (PME) [42] method with default Ewald parameters and the van der Waals force was truncated at 10 Å with energy shift.

### Trajectory and structure analysis

PTRAJ program in AMBER 11 was used in trajectory analysis. The solvent accessible surface area (SASA) was calculated using the NACCESS program [43]. The RMSD values are calculated through all heavy atoms relative to the NMR structure. The first 1.0 ns trajectories were ignored in the analyses to allow for structural equilibrium under solvation simulations. The MMPBSA program in AMBER 11 was used to calculate the binding free energy between residues. The fraction of native contacts (NC for short below) was used as a measure of structure features during the simulations. The native contact was defined using a 7 Å distance cutoff which can represent both base-pairing interactions as well as base stacking between neighboring bases. The free energy landscape (potential of mean force (PMF) profiles) is calculated using the algorithm proposed by Pande et al.[44], in which the free energy is defined as –K_B_T*ln*(NR/NT), where NR is the number of structures in each counted region defined by the order parameters, fractions of native contacts of P1-L1-L2 and P3-L3, NT is the total number of the structures. The trajectory visualization and figures were generated using VMD [45], UCSF Chimera packages [46] and the PyMOL Molecular Graphics System, Version 1.3, Schrödinger, LLC.

## Results

Our aim is to look for the possible stable structure of ligand-free aptamer of *Bsu* preQ_1_ riboswitch that can sense the ligand preQ_1_. Fig. 1 displays the secondary and tertiary structures of the ligand-bound aptamer domain of *Bsu* preQ_1_ riboswitch (PDB ID: 2L1V [27]). The ligand-bound aptamer adopts an H-type pseudoknot with two stems (P1 and P2) and three loops (L1, L2 and L3). The stem P2 stacks above the P1. L1 and L3 lie in the major and minor grooves of P2 and P1, respectively. The L2 loop is unusual 6-nt long and lies in the minor groove of P2. We have performed three (two 75 ns and one 600 ns) unfolding simulations started from the experimental NMR bound structure with removal of preQ_1_ to look for the stable states of preQ_1_-free aptamer. For comparison, we also performed similar three simulations on the ligand-bound aptamer.

### Ligand-free aptamer has a stable state with a folded P1-L3 structure

**Figure 2:**
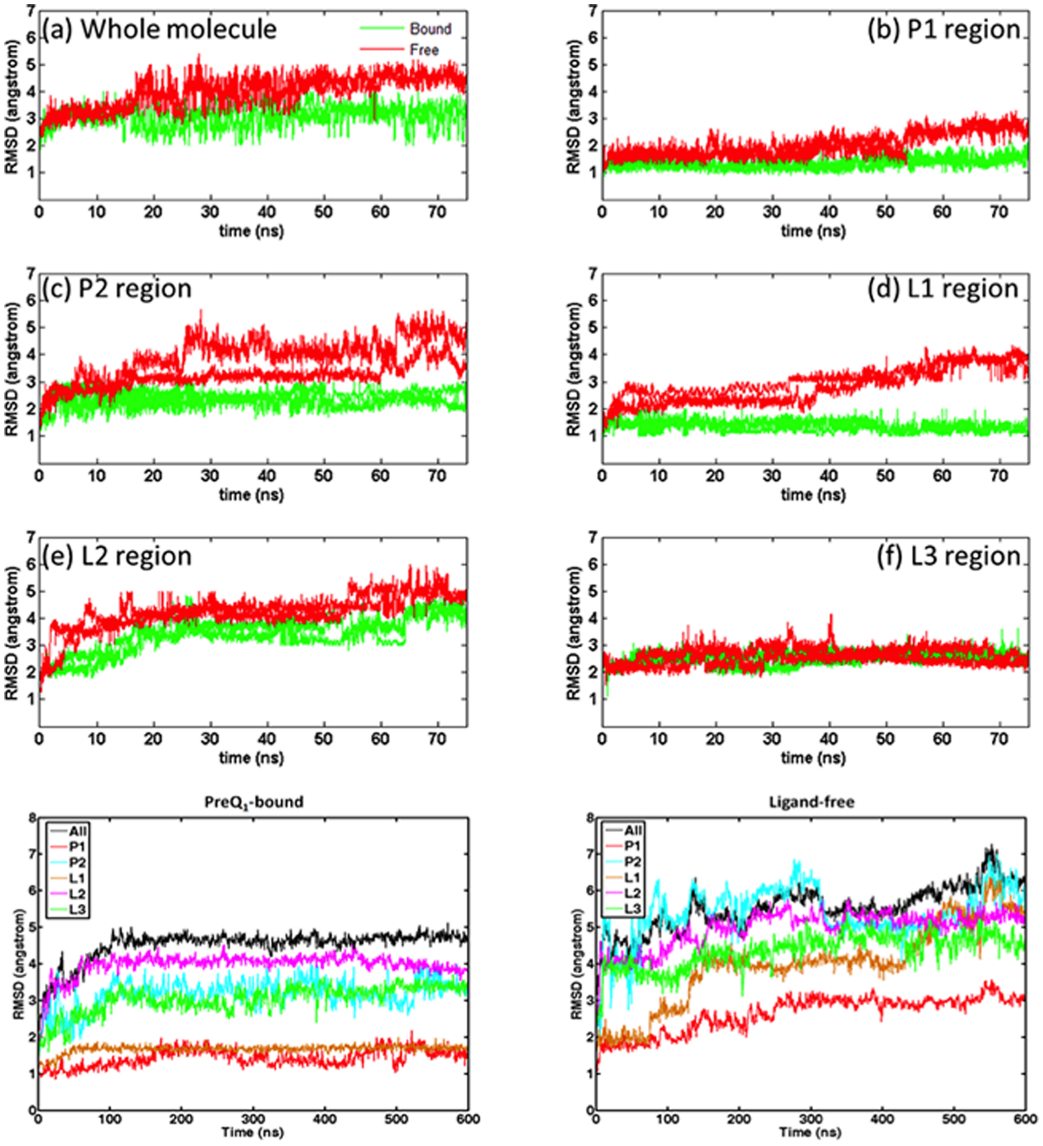
Heavy atom RMSD of the preQ_1_-bound and ligand-free aptamer simulations. The top six subfigures are from the 75_1_-bound (green) and ligand-free (red). The bottom two subfigures are from the 600 ns simulations with each line represents a fragment as indicated in figure legends.

The simulation results show that the bound structure with the removal of the ligand is not stable and it evolves into a conformation with a stable P1-L3 structure. Fig. 2 shows the heavy atom RMSDs of the simulated snapshots relative to the NMR structure of the whole aptamer as well as different parts (P1, P2, L1, L2 and L3). The averaged results are summarized in Table 1. It can be seen that the aptamer behaved consistently in all these trajectories after removal of the ligand. Likewise, the dynamics were highly similar when preQ_1_ was bound. Overall, the heavy atom RMSD of the whole molecule remained at the level of about 4 Å when preQ_1_ was bound and increased to an average of about 5.6 Å when preQ_1_ was removed. Among the fragments, P1 and L3 exhibited the highest stability in both bound and unbound simulations. The main conformational changes of the aptamer from the bound to free states are from L1 and P2 regions. L1 shows comparable stability to P1 in the preQ_1_-bound simulations and its average RMSD maintained in the range of 1.3 Å to 1.6 Å throughout but increased to 4.15 Å in the unbound simulations. Further analysis revealed that the enhanced fluctuation of loop L1 in the unbound simulations is attributable to the loss of the hydrogen bond network formed around preQ_1_. From the bound to free states, the average RMSD of P2 region changes from 3.25 Å to 5.41 Å. L2 region shows the largest movement in both free and preQ_1_-bound simulations. This is because its lack of close contacts with other parts of the molecules, consistent with the observation of Zhang et al. [47] who suggested that L2 is the only highly dynamic region of the preQ_1_-bound aptamer.

**Table 1:** Average RMSD of the overall structure and different regions.

Regions	preQ_1_-Bound		Ligand-Free	
	75 ns	600 ns	75 ns	600 ns
Whole molecule	3.10 (0.35)	4.12 (0.22)	4.08 (0.58)	5.60 (0.63)
L1	1.36 (0.16)	1.53 (0.13)	2.83 (0.61)	4.15(1.15)
L2	3.38 (0.67)	4.07 (0.37)	4.17 (0.66)	4.99 (0.49)
L3	2.43 (0.20)	3.04 (0.25)	2.77 (0.27)	4.36 (0.46)
P1	1.36 (0.18)	1.59 (0.11)	2.03 (0.42)	2.68 (0.48)
P2	2.34 (0.23)	3.25 (0.16)	3.96 (0.76)	5.41 (0.72)

Numbers in parenthesis are standard deviations. Units are in Å.

**Figure 3:**
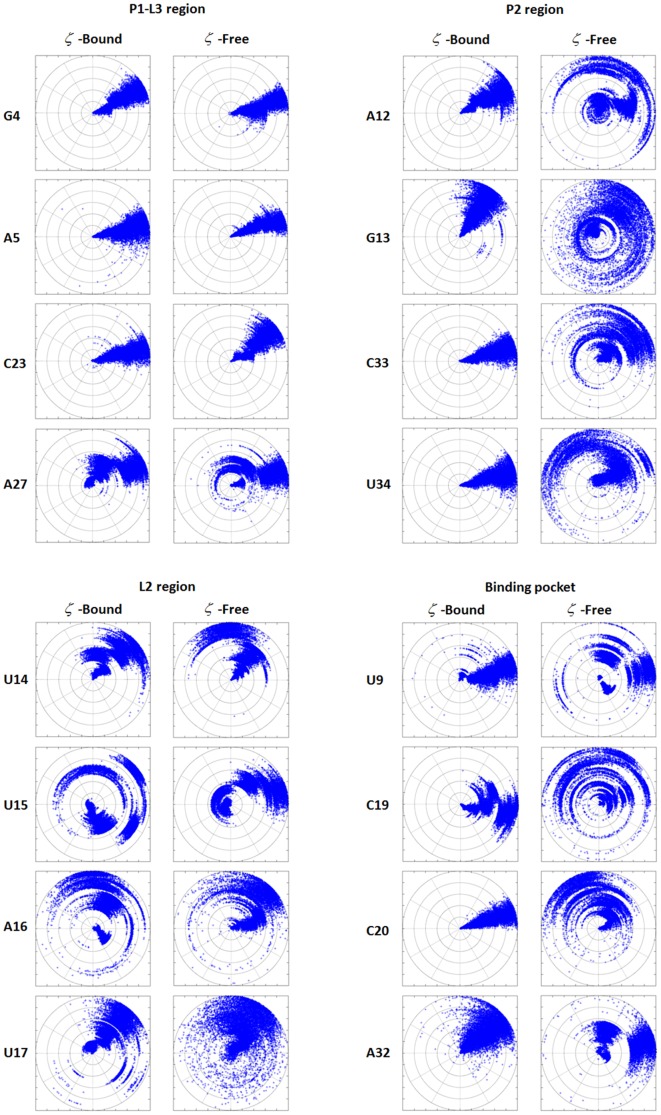
Torsion angles ζ during the 600 ns ff98 simulations. The radial axes are the times with the origin as 0π in anticlockwise direction with 0 being at the right side of the plot.

The conformational variations of the aptamer from the bound state to the free state can be further examined using the ζ torsion angles of the key nucleotides. Torsion angle ζ is considered one of the most significant indicators to the conformational variability observed in RNA molecules [14], [48]. Figure 3 shows that the ζ angles of the key nucleotides in P1-L3 and L2 regions fluctuate similarly in both preQ_1_-bound and ligand-free simulations. In P1-L3 region, the ζ angle fluctuated within 45 degrees (upper left in Figure 3). Considerably larger degree of fluctuations is evident in L2 region in both preQ_1_-bound and ligand-free simulations that ranged from more than 180 degrees to 360 degrees (lower-left in Figure 3). This is consistent with the observed large heavy atom RMSD relative to the NMR structure. In contrast, ζ angles in the P2 region and binding pocket fluctuated notably differently in the preQ_1_-bound and ligand-free simulation. The ζ angles of the key nucleotides in pseudoknot and binding pocket fluctuated within typically 45 degrees in preQ_1_-bound simulation, while they show much larger fluctuations in ligand-free simulation (right side in Figure 3). These observations again show that the P1-L3 region is stable independent of ligand binding, the L2 region is inherently mobile in both cases, and the P2 region and binding pocket are stable in preQ_1_-bound state but lost their native conformations in ligand-free state.

**Figure 4:**
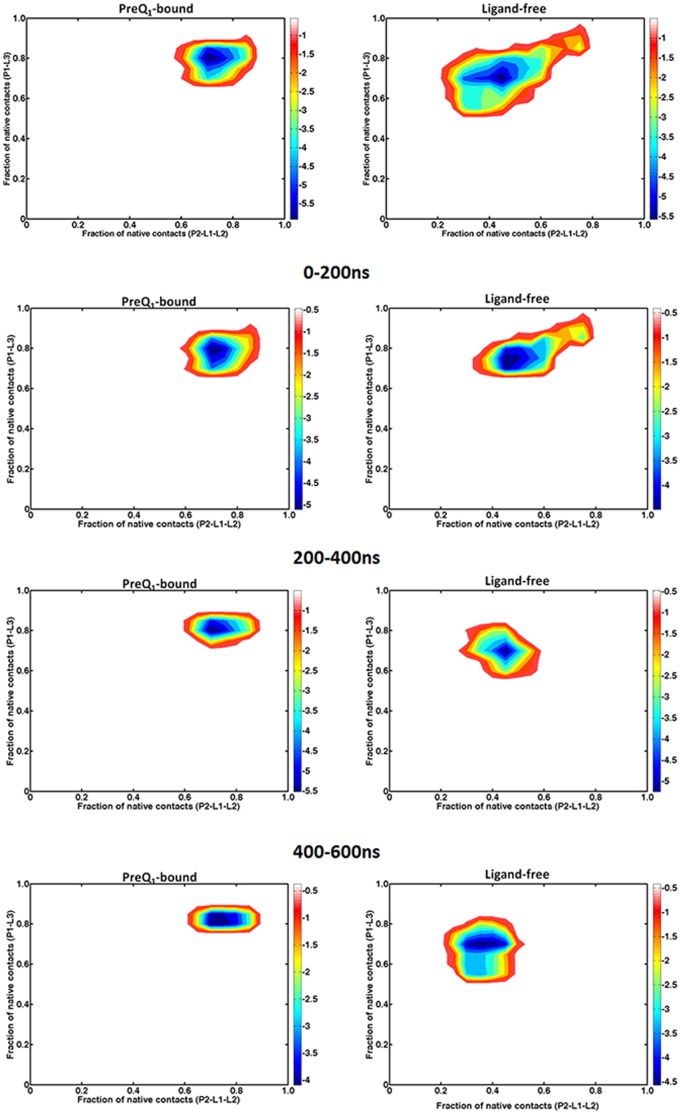
Two-dimensional free energy landscapes from the 600 ns simulations and those in different time frames of preQ_1_–bound (left) and ligand-free (right). The order parameters are the fractions of native contacts for P1-L3 and P2-L1-L2, respectively.

To further examine the stability of the pseudoknot structure without the preQ_1_, two-dimensional potential of mean force (PMF) profiles were built from the 600 ns trajectories with and without the ligand (Fig. 4), using the fraction of native contacts (NC) in P1-L3 and P2-L1-L2 as the coordinates, respectively. To examine the consistency of the simulations, we also built the PMF profiles from the 600 ns simulations in different time frames (Fig. 4). In the presence of preQ1 ligand the aptamer stayed mostly in a state with 80% NC of P1-L3 and P2-L1-L2. This was true for different time windows of the 600 ns simulation. The 20% NC loss was due to the flexible residues located in terminal and L2 region. In the absence of ligand, the PMF profiles for the first 200 ns includes a minor basin located at around 80% NC of P1-L3 and P2-L1-L2 and a major basin at 70% NC of P1-L3 and 50% NC of P2-L1-L2 (Fig. 4). As simulation time progressed, the minor basin disappeared and the major basin shifted towards fewer NC of P2-L1-L3 whereas NC of P1-L3 remained similar. In the time window of 400–600 ns, the major basin shifted to around 70% NC of P1-L3 and 40% NC of P2-L1-L2. Thus, P2-L1-L2 fragments were notably less stable than P1-L3. The stable P1-L3 structure was due to the special triplex conformation called “A-amino kissing motif”. However, the collapse of the binding pocket also affects the top of P1-L3 triplex and about 10% NC of P1-L3 was lost compared to the PMF basin of the preQ1-bound aptamer. The results above indicate that the major difference between the preQ1-bound and ligand-free stable structures of the aptamer comes from the binding pocket and P2 regions while the P1-L3 triplex remains stable in both cases.

### Opening of the binding pocket in the ligand-free simulations

**Figure 5:**
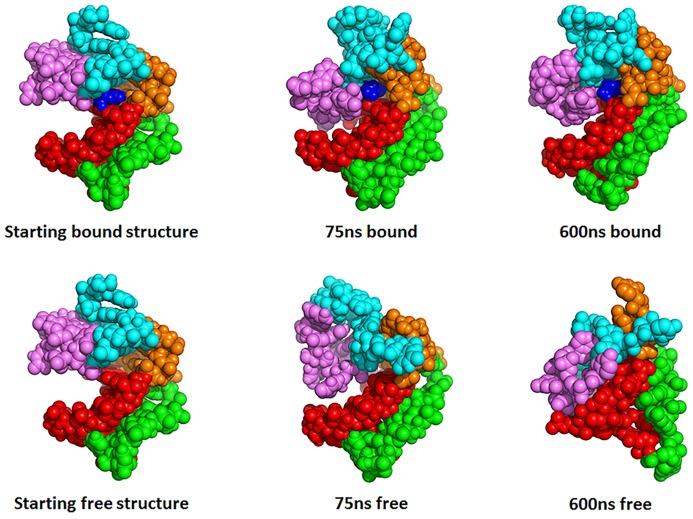
Van der Waals representations of the last structures from the 75_1_-bound (upper middle), 75 ns ligand-free (lower middle), 600 ns preQ_1_-bound (upper right), 600 ns ligand-free (lower right) simulations. Structural elements are colored light pink (P1), cyan (P2), orange (L1), violet (L2), green (L3) and blue (preQ_1_), respectively.

**Figure 6:**
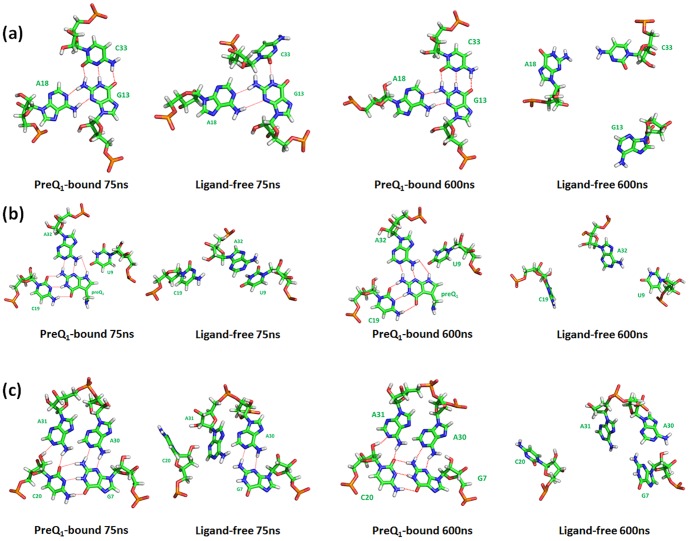
Key structural features in the preQ_1_-bound and ligand-free simulations at different times. Hydrogen bonds are showed using red dashed line. (a) P2-L2 base triplet; (b) preQ_1_ base quadruplet; (c) P1-L3 base quadruplet.

The NMR structure of the ligand-bound preQ_1_ riboswitch aptamer shows that the ligand preQ_1_ is located in a closed binding pocket (Fig. 5) [27]. Also shown in Fig. 5 are the end structures observed in the 75 ns and 600 ns preQ1-bound and ligand-free simulations. The bound preQ_1_ directly forms a Watson-Crick pair with C19 and hydrogen bonds with A32 and U9 and they form a base quadruplet (Fig. 6b). This preQ_1_ base quadruplet is sandwiched between and stabilized by a P2-L2 base triplet (C33-G13-A18) (Fig. 6a) and a P1-L3 base quadruplet (Fig. 6c). The latter P1-L3 base quadruplet is formed by the base pair G7∶C20 at the top of P1 and two contiguous nucleotides A30 and A31 in L3 region, which form an unusual A-platform in the minor groove of P1.

**Figure 7:**
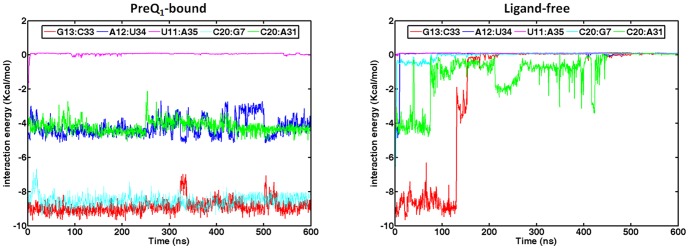
Interaction energies of key residues located in pseudoknot and binding pocket in the 600

**Figure 8:**
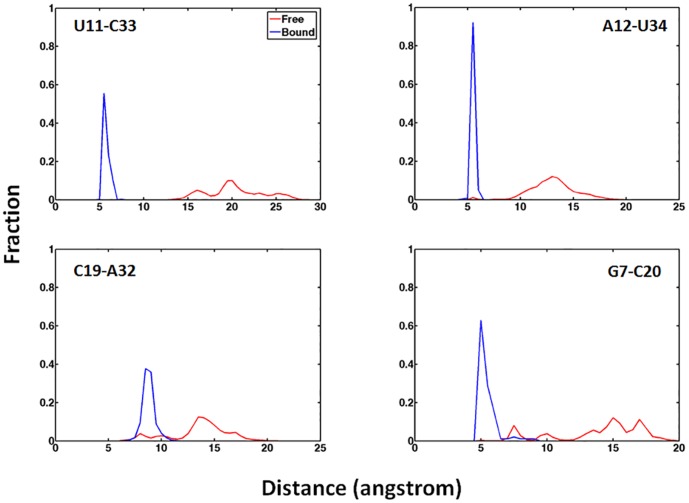
Histograms of the distances between the centers of mass of the key residues from the 600

The key local structures in binding pocket were rather stable throughout both 75 ns and 600 ns simulations when preQ_1_ was bound. In contrast, these structures became considerably less stable when preQ_1_ was removed and started to rapture even as early as in 75 ns and became completely disrupted by the end of 600 ns simulation (Fig. 5). The pairwise interaction energies of key residues located in binding pocket are shown in Figure 7. The interaction energies of A31-C20-G7 around binding pocket remained steady during the 600 ns preQ_1_-bound simulation, which indicates these regions were stable in the presence of the ligand. In contrast, without the ligand, the interaction energies in binding pocket all rose to 0 kcal/mol level after 200 ns. Figure 8 shows the histograms of the distances between centers of mass of the key residues. It can be seen that the contacts between the bases in binding pocket (C19∶A32 and G7∶C20) were well maintained throughout in the preQ_1_-bound simulation. However, without the ligand, the centers of mass sampled much wider range and longer distances during the simulation, indicating that the nucleotides in binding pocket moved apart from each other. Fig. 7 also indicates that the completely breaking of P2 helix in ligand-free aptamer is after the opening of the binding pocket. The only exception was the interaction between U11 and A35 that increased to around 0 kcal/mol in short time during both ligand-free and ligand-bound simulations, indicating the flexibility of terminal residues.

We are also interested in the dynamics of C19 since C19 forms Watson-Crick pair with preQ_1_ in the bound structure and was suggested to be the initial recognizing site by preQ_1_ [27]. At 600 ns, A18, C19 and C20 all moved outward and became more exposed to solvent, as indicated by the larger SASA shown in Table 2. The outward movement of C19, in particular, opened up a channel in the major groove side that may allow preQ_1_ to move into the binding pocket(Fig. 5 and Fig. 6). It is noted that the major groove side of P1 is also the only place where preQ_1_ can be accessed from outside in the ligand-bound structure [27]. In the opposite side of the major groove of P1 the binding pocket also opens around C20 but the size of the entrance is too small that impedes the movement of preQ_1_ into the binding pocket. Thus, although both sides are possible, the simulation suggests that the major groove side is more likely the ligand entrance site.

**Table 2:** Solvent accessible surface area (in Å^2^) of key nucleotides related to the ligand binding.

Base ID	NMR structure	preQ_1_-bound		Ligand-free	
		75 ns	600 ns	75 ns	600 ns
A18	107	106	110	139	147
C19	154	151	158	196	213
C20	133	135	137	202	215
C21	103	103	105	114	111

**Figure 9:**
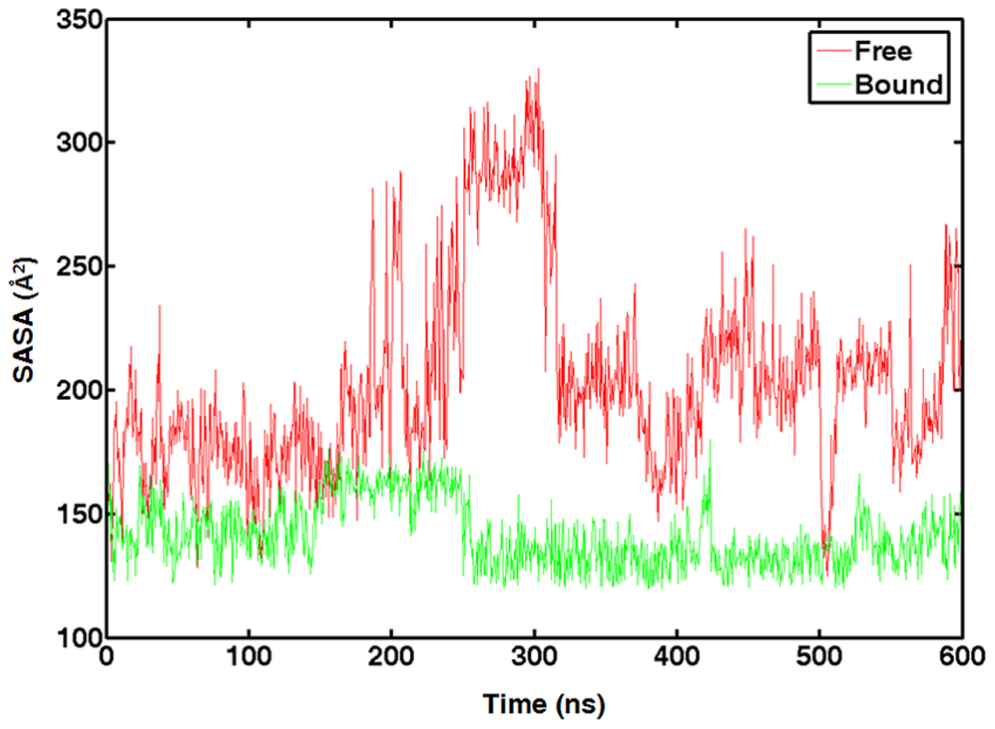
Solvent accessible surface area of C19 in the preQ_1_-bound and ligand-free aptamer 600 ns simulations.

**Figure 10:**
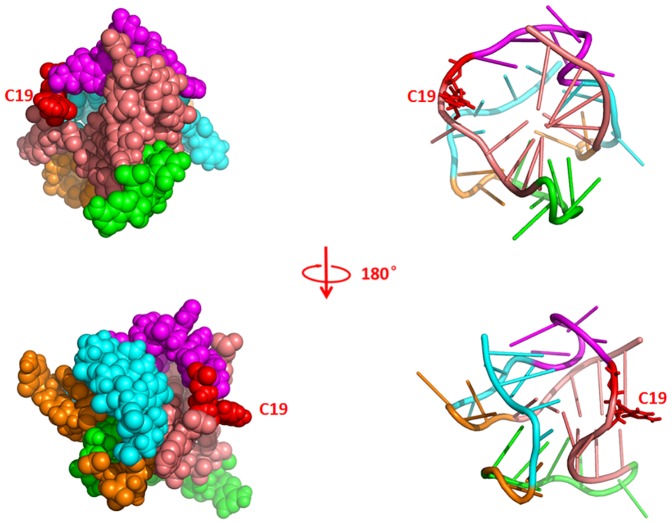
Van der Waals and cartoon representations of the last snapshot taken from the 600-free trajectory. Structural elements are colored light red (P1), cyan (P2), orange (L1), violet (L2), green (L3) and red (C19), respectively. It shows open binding pocket (next to C19).

During simulation, C19 oscillates between close and open positions (Fig. 3). This dynamic behavior of C19 is clearly demonstrated by the time variation of its SASA (Fig. 9). At the beginning of the simulation, C19 was deeply buried and its SASA was about 150 Å^2^, close to the value found in the NMR structure (Table 2), and soon reached level close to 200 Å^2^. In the entire 600 ns simulation SASA of C19 experienced large degree of fluctuation, to as high as 300 Å^2^, more than double of that in the NMR structure. It reached 213 Å^2^ at the end of the simulation. In comparison, when preQ1 was present, the SASA of C19 fluctuated between 130 Å^2^ and 170 Å^2^ and reached 158 Å^2^ at the end of the 600 ns simulation, comparable to that found in the NMR structure. The outward movement of C19 makes it as a potential candidate of the initial recognizing site by preQ1 (Fig. 10).

**Figure 11:**
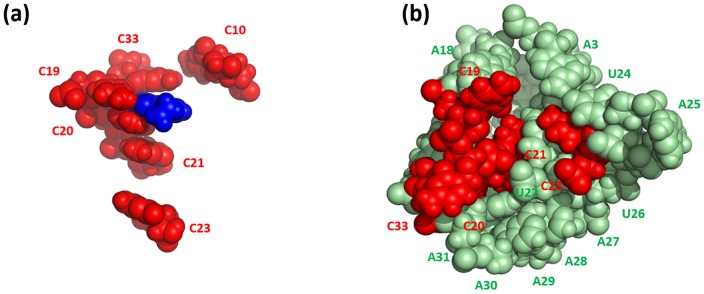
Illustration of the cytosine clusters. (a) The six cytosines and preQ_1_ in the preQ1-bound structure. (b) Cytosines (red) in the entrance of the opened binding pocket in the ligand-free simulation at 600 ns.

Finally, we found that the opened binding pocket in the major groove side of P1 forms a C-rich pool that may assist the binding of preQ_1_. Figure 11a illustrates the relative positions of all cytosine nucleotides and preQ_1_ in the ligand-bound structure. It can be seen that preQ_1_ is enclosed by four out of six cytosines (C19, C20, C21, and C33). What is more interesting is that in the opened binding pocket five (above four plus C23) of the six cytosines are located around the entrance (Figure 11b) and form a C-rich pool, even though C23 is not in direct contact with preQ_1_ in the preQ_1_-bound structure. We propose that the presence of cytosine cluster around the preQ_1_ entrance may facilitate the binding of preQ_1_. Because PreQ_1_ molecule is structurally similar to guanine, cytosines may act as intermediaries that help to capture preQ_1_ and direct it towards the binding site by forming interactions.

## Discussion

Our simulation results indicate that the ligand-free aptamer of the *Bsu* preQ_1_ riboswitch has a stable conformation with open binding pocket. In such a conformation the pseudoknot does not exist but P1 and L3 forms a stable triplex conformation as that in the preQ1-bound structure. This is in agreement with the results of Suddala et al [31] who found that the ligand-free aptamer of the *Bsu* preQ_1_ riboswitch has an ensemble of pre-folded conformations wherein their A-rich 3′ tail (L3) adopts transient interactions with the P1-L1 stem-loop. This conformation is also similar to the stable intermediate state found in our previous paper [30]. However, since it is difficult to simulate long-time dynamics at all-atom level, we didn't see the possible transitions from this state to other states, e.g., the state with P1-L1 stem-loop or the ligand-bound-like state.

By the way, Suddala et al. [31] also showed that the ligand-free aptamer of the *Tte* preQ_1_ riboswitch has a similar ensemble of pre-folded conformations as that of *Bsu* preQ_1_ riboswitch. But we previously found [30] that the ligand-free aptamer of the *Tte* preQ_1_ riboswitch has a stable intermediate state which has a structure that is very close to the ligand-bound one but the P2 helix only has one of the base pairs G11∶C30 remained and the base pairs C9∶G33 has broken. This is very similar to the structure (P-fold) of the free aptamer of the *Fnu* preQ1 class I riboswitch that preorganizes into a partially formed pseudoknot fold [29]. Despite this difference, all these results indicate a folded P1-L3 conformation in the structure of the ligand-free aptamer of the *Tte* preQ_1_ riboswitch.

Beside the global structure of the ligand-free aptamer of *Bsu* preQ_1_ riboswitch, our all-atom simulations can also reveal some features of its binding pocket, including its opening, the oscillating behaviors of C19 and the C-rich pool in the opened binding pocket. The opening of the binding pocket opens up a channel in the major groove side that allows preQ_1_ to move into the binding pocket. The outward-inward movement of C19 makes it as a potential candidate of the initial recognizing site by preQ_1_. The presence of cytosine cluster around the preQ_1_ entrance may facilitate the binding of preQ_1_.

Cations and anions may play critical roles in RNA dynamics. For simulating these roles realistically, both of them should be considered. Since only Mg^2+^ is considered in our simulations, to assess the salt effect, we performed additional 100 ns preQ_1_-bound and ligand-free simulations with 0.3 M NaCl in addition to the 0.15 M Mg^2+^. The results are shown in Figure S1. Overall, addition of 0.3 M NaCl did not change the dynamics significantly and the RMSDs remained at the levels similar to those observed in simulations with only Mg^2+^ ions and without NaCl. The observed dynamics in these two types of simulations were highly consistent. Taken together, we conclude that inclusion on NaCl in addition to neutralizing Mg^2+^ ions is helpful to stabilize the structure.

We also examined the effect of the force field on our results. We performed a comparative simulations using Amber ff99bsc0 since it made a key modification on the γ torsion angles [49], [50]. In terms of RMSD, we found that the two types (Amber ff98 and ff99bsc0) of simulations were highly consistent (Figure S1) when they were performed with 0.15 M Mg^2+^ and 0.3 M NaCl. The overall RMSD remained at around 4 Å within the first 100 ns. Furthermore, the γ and χ torsion angle distributions from the simulations are shown in Figure S2 and are compared to those found from the NMR structure. The γ torsion angles distributed around g+ and anti-regions and lacked sampling of the area between these two regions. In contrast, the ff99bsc0 appears to have strong bias towards g+ region. On the other hand, the χ torsion angle distributions of ff98 and ff99bsc0 appear to resemble the one from the NMR structure. This is encouraging. It is also evident that, with the same force field, the distributions from the ligand-free simulations were highly similar to those in the preQ_1_-bound simulations. This is interesting because, even though the γ and χ distributions with the same force field show close resemblance between the ligand-free and preQ_1_-bound simulations, the dynamics exhibited notable differences. Furthermore, the dynamics observed in the preQ_1_-bound simulations was qualitatively similar with different force fields. This was also true for the ligand-free simulations. Thus, we conclude that the elevated dynamics in the ligand-free simulations was unrelated to the differences between the force fields. The consistency also marginally enhances our confidence in the results.

**Figure 12:**
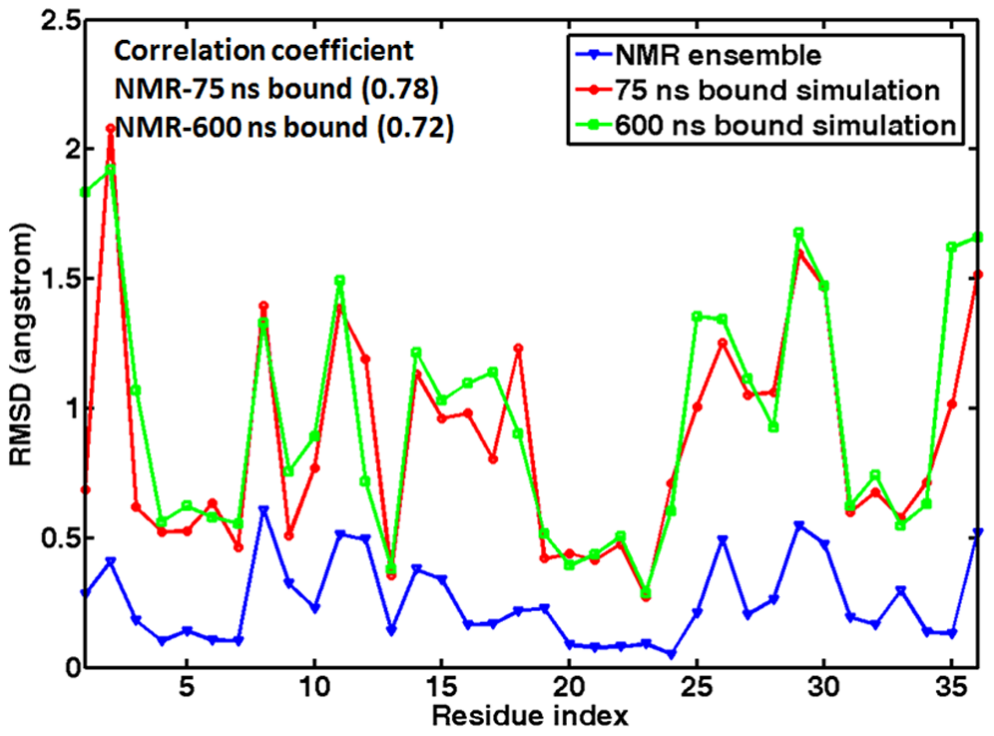
Average heavy atom RMSD for each nucleotide with respect to the first NMR structure in the 75-bound simulations (blue), 600 ns preQ1-bound simulation (green), and NMR ensemble (red).

The reliability of the simulated dynamics using Amber ff98 can also be shown by the average heavy atom RMSDs of the individual nucleotides relative to the NMR structures that were calculated after rigid body alignment of the entire molecule (Figure 12). For comparison, the RMSDs from the NMR structures are also shown in Figure 12. The RMSDs calculated from preQ_1_-bound simulations closely resemble that calculated from NMR ensemble with the simulations showing higher degree of fluctuation. This difference is likely attributable to the tight ensemble observed in NMR structures. Nevertheless, the correlation coefficients between the simulated and NMR RMSDs were 0.78 for the two 75 ns simulations and 0.72 for the 600 ns simulation. Such a level of correlation indicates that the simulations captured the key features of the dynamics of the preQ_1_-bound state.

## Conclusion

MD simulations have been performed for the aptamer domain of the *Bsu* preQ_1_ riboswitch on both preQ_1_-bound and ligand-free structures. The results indicate that preQ_1_-ligand plays important roles in stabilizing the overall structure. In the absence of preQ_1_, the aptamer moved away from the starting NMR structure and formed a relatively stable and compact conformation with open binding pocket. In such a conformation the pseudoknot does not exist but P1 and L3 forms a stable triplex conformation or “A-amino kissing motif” as that in the preQ_1_-bound structure. There are five cytosine's located close to the entrance of the open binding pocket that form a Cyt-rich pool. This conformation may be a candidate for the initial binding of preQ_1_. The simulations further suggested a multi-step process in which the ligand initially enters the open binding pocket and the entrance is subsequently closed by nucleotide C19. These results can help to understand the details of the preQ_1_ binding process.

## Supporting Information

Figure S1 **Heavy atom RMSD in preQ_1_-bound and ligand-free simulations with 0.3 M NaCl and 0.15 M Mg^2+^ and with different force fields, respectively.** Different colors are used to represent different parts of the aptamer.
(TIF)

Figure S2 **Distributions of γ and χ torsion angles.** Top two: native preQ_1_ riboswitch aptamer observed in the NMR structure. Middle four: from preQ_1_-bound and ligand-free 600 ns ff98 simulations. Lower four: from preQ1-bound and ligand-free 200 ns ff99bsc0 simulations.
(TIF)
