# Supplementary figures and images for: Insights into Ligand Binding to PreQ1 Riboswitch Aptamer from Molecular Dynamics Simulations
Source: PLoS One. 2014 Mar 24;9(3):e92247. doi: 10.1371/journal.pone.0092247 (PMC3963873; doi:10.1371/journal.pone.0092247)

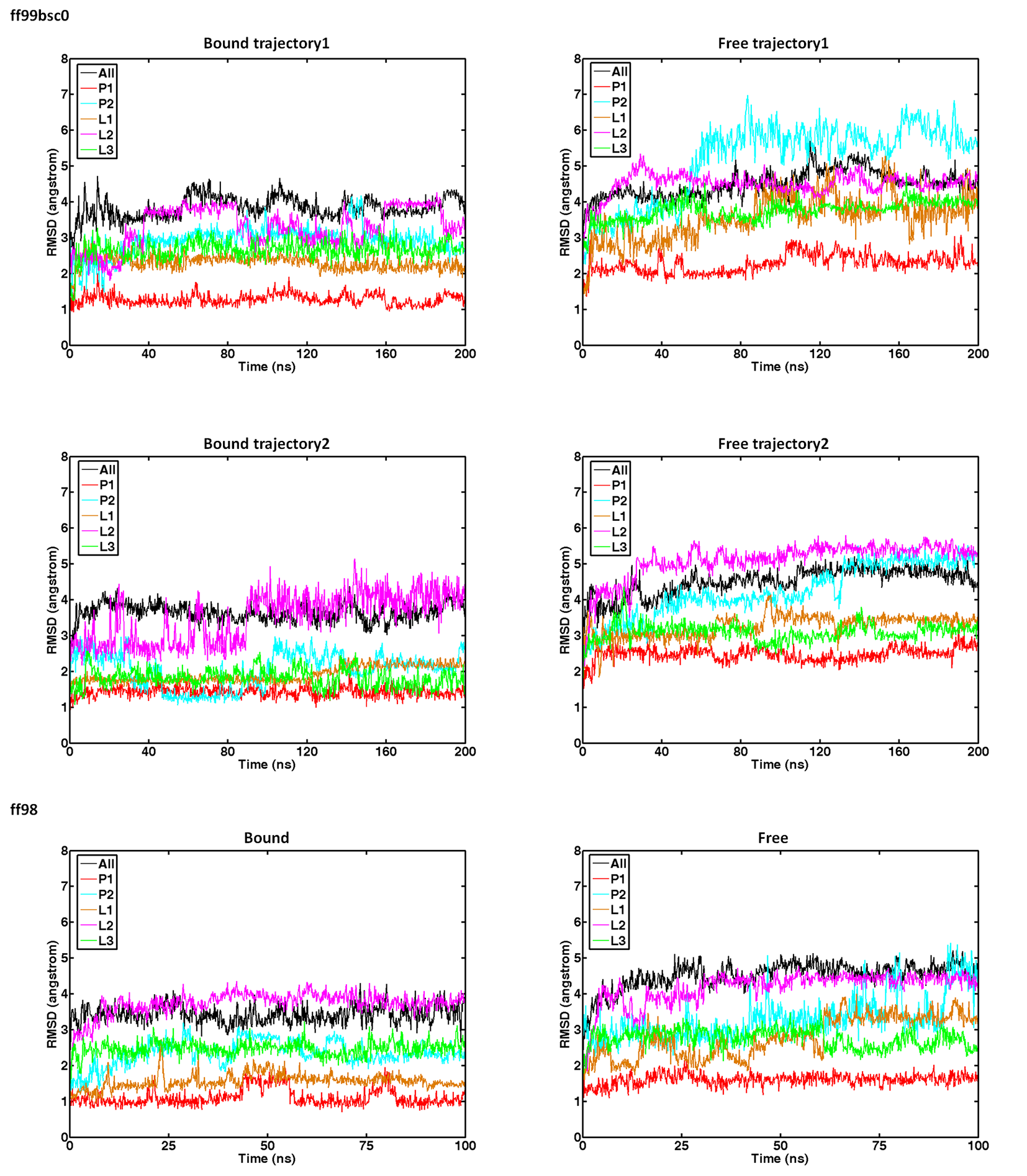

Supplement: Figure S1 — Heavy atom RMSD in preQ1-bound and ligand-free simulations with 0.3 M NaCl and 0.15 M Mg2+ and with different force fields, respectively. Different colors are used to represent different parts of the aptamer. (TIF) [file pone.0092247.s001.tif]

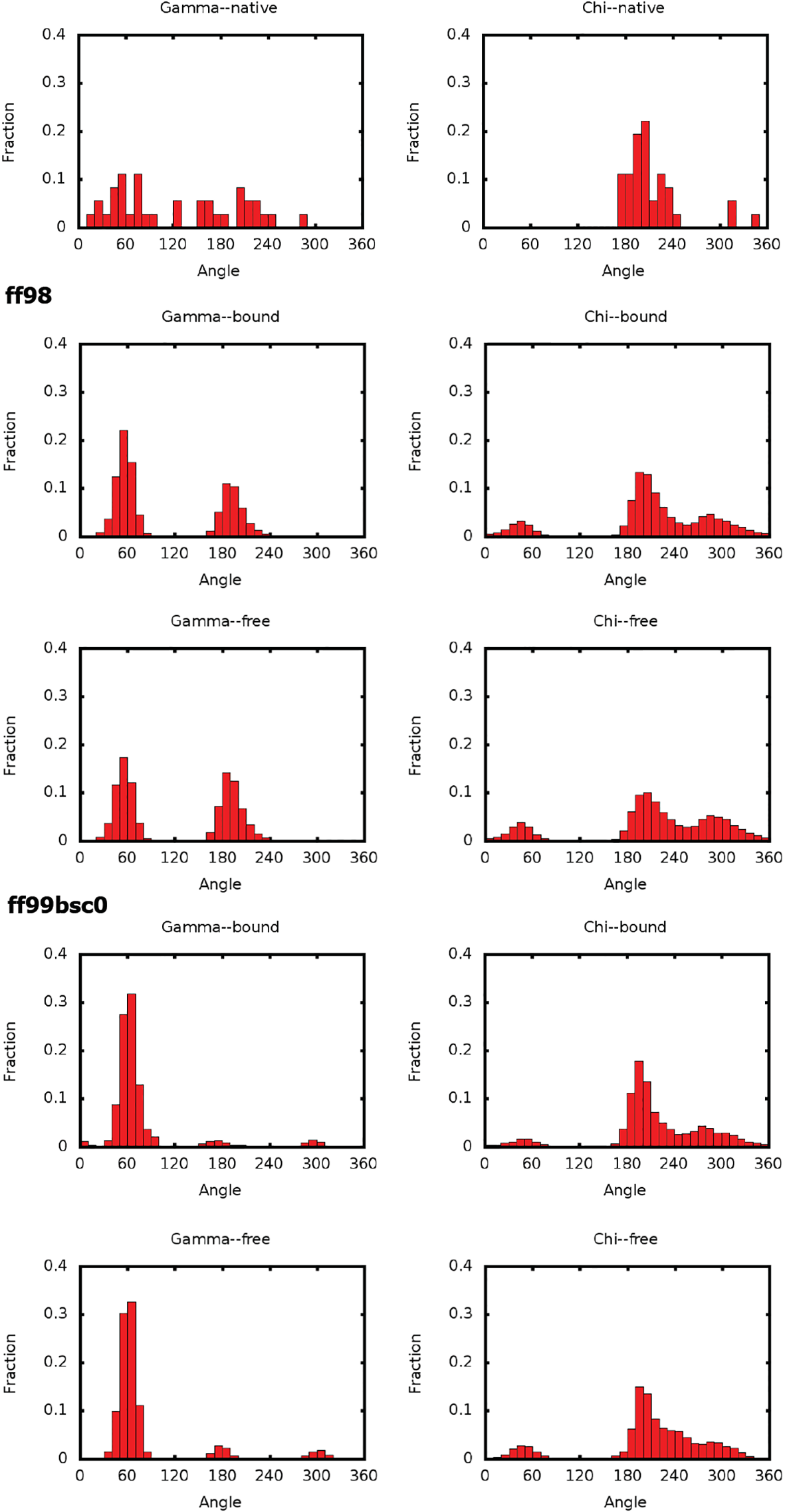

Supplement: Figure S2 — Distributions of γ and χ torsion angles. Top two: native preQ1 riboswitch aptamer observed in the NMR structure. Middle four: from preQ1-bound and ligand-free 600 ns ff98 simulations. Lower four: from preQ1-bound and ligand-free 200 ns ff99bsc0 simulations. (TIF) [file pone.0092247.s002.tif]
